# Associations of Chronic Lymphocytic Leukemia (CLL) Susceptibility Variants Across the CLL Etiologic Continuum

**DOI:** 10.3390/cancers18213556

**Published:** 2026-11-05

**Authors:** Raphael Mwangi, Geffen Kleinstern, Dennis Robinson, Aaron D. Norman, Kari G. Rabe, Janet E. Olson, Yuan Yao, Nicholas J. Boddicker, Rosalie Griffin, Esteban Braggio, James R. Cerhan, Curtis A. Hanson, Celine M. Vachon, Sameer A. Parikh, Tait D. Shanafelt, Susan L. Slager

**Affiliations:** 1 Division of Computational Biology, Mayo Clinic, Rochester, MN 55902, USA; mwangi.raphael@mayo.edu (R.M.);; 2 Division of Clinical Trials and Biostatistics, Mayo Clinic, Rochester, MN 55902, USA; 3 Faculty of Social Welfare & Health Sciences, School of Public Health, University of Haifa, Haifa 3498838, Israel; 4 Division of Epidemiology, Mayo Clinic, Rochester, MN 55902, USAcerhan.james@mayo.edu (J.R.C.);; 5 Division of Hematology, Mayo Clinic, Rochester, MN 55902, USA; 6 Department of Epidemiology, The University of Texas MD Anderson Cancer Center, Houston, TX 77030, USA; 7 Department of Lymphoma & Myeloma, The University of Texas MD Anderson Cancer Center, Houston, TX 77030, USA; 8 Department of Hematology and Oncology, Mayo Clinic, Phoenix, AZ 85054, USA; 9 Department of Laboratory Medicine and Pathology, Mayo Clinic, Rochester, MN 55902, USA; 10 Department of Medicine, Division of Hematology, Stanford University, Stanford, CA 94305, USA

**Keywords:** chronic lymphocytic leukemia, monoclonal B-cell lymphocytosis, susceptibility variants, polygenic risk score

## Abstract

**Background:** Low-count monoclonal B-cell lymphocytosis (LC-MBL) is a precursor to chronic lymphocytic leukemia (CLL). Forty-one inherited susceptibility variants for CLL have been reported, but whether these variants impact CLL, through the initiation of the clone (LC-MBL) or progression of LC-MBL to CLL, is not well characterized. **Methods:** We evaluated 41 previously reported CLL susceptibility variants and the CLL polygenic risk score (CLL-PRS, a weighted score of the 41 variants) among a study of 8322 individuals who screened negative for MBL based on eight-color flow cytometry, 1601 individuals who screened positive for LC-MBL, and 2606 CLL patients. Logistic regression was used to estimate odds ratios (ORs) and 95% confidence intervals (CI) for LC-MBL compared with MBL-negative individuals and for CLL compared with LC-MBL (herein labeled progression). Models were adjusted for age, sex, and genetic principal components. **Results:** Of the 41 variants, 22 (54%) were associated with LC-MBL and 20 (49%) with progression (all *p* < 0.05). The ORs for eight variants differed between LC-MBL risk and progression (false discovery rate, FDR < 0.05), including two (rs6708784, 2q13; rs391855, 16q24.1) with associations in opposite directions. The CLL-PRS was associated with both LC-MBL (OR = 1.77, 95% CI: 1.66–1.88, *p* = 3.7 × 10^−68^) and progression (OR = 1.45, 95% CI: 1.35–1.56, *p* = 8.1 × 10^−24^). **Conclusions:** The effects of individual CLL-risk variants vary across the etiological continuum of disease with a subset associated with clonal initiation of LC-MBL and clonal progression to different degrees, or even in opposite directions. The CLL-PRS may help identify individuals at elevated risk for both LC-MBL and progression to CLL.

## Simple Summary

Chronic lymphocytic leukemia (CLL) is a common blood cancer. It is preceded by a condition, called monoclonal B-cell lymphocytosis (MBL), in which a small population of clonal B-cells circulates in the blood. Although most people with MBL never progress to CLL, it is unknown why some do. Earlier studies found 41 inherited genetic variants that increase the risk of CLL. Using a study of more than 12,000 people, including people without MBL, people with MBL, and people with CLL, we evaluated the impact of these inherited variants in developing MBL and progressing from MBL to CLL. We found some variants linked more strongly to developing MBL and others to progression, and a few variants appeared to act in opposite directions. A combined genetic risk score of these variants was linked to both developing MBL and progression. These findings can guide future studies of who is most likely to develop CLL.

## 1. Introduction

Monoclonal B-cell lymphocytosis (MBL) is a common condition and a precursor to chronic lymphocytic leukemia (CLL) [1,2,3]. MBL is categorized as low-count (LC-MBL; <0.5 × 10^9^/L) or high-count (HC-MBL; 0.5 to <5 × 10^9^/L) based on the absolute clonal B-cell count [4]. Individuals with HC-MBL progress to CLL requiring therapy at a rate of approximately 1–2% per year [5]. LC-MBL is detected in approximately 5–18% of adults over age 40 screened by flow cytometry [6,7,8]. Although most individuals with LC-MBL do not progress to CLL, recent studies from our group have demonstrated that LC-MBL is associated with a 2.6-fold increased risk of lymphoid malignancy [9], a 1.9-fold risk of melanoma [10], and a 1.6-fold risk of serious infections [11]. MBL, CLL, and outcomes after CLL diagnosis, such as time to first treatment and infection [12,13], can be viewed as a continuum of B-cell malignancy, and biomarkers are needed to identify individuals at risk at each point along this continuum.

Genome-wide association studies (GWAS) have identified 41 common single nucleotide polymorphisms (SNPs) associated with CLL risk [14,15,16,17,18,19,20], many of which map to loci implicated in immune regulation [15]. When combined into a polygenic risk score (PRS), the CLL-PRS is strongly and robustly associated with CLL risk, independent of family history of hematological cancer [21,22]. The CLL-PRS has also been found to be associated with risk of MBL (including both LC- and HC-MBL) and 21 of the 41 SNPs were individually associated with MBL [22,23,24]. However, it remains unclear whether the associations of these SNPs differ between risk of LC-MBL and progression from LC-MBL to CLL. We assembled a cohort of individuals spanning the etiologic continuum from those who screened negative for MBL to those with LC-MBL and overt CLL to evaluate the effect of the 41 CLL-risk variants and the CLL-PRS.

## 2. Methods

### 2.1. Study Population

This study was approved by the Mayo Clinic Institutional Review Board, with all participants providing informed written consent. Individuals screened for MBL were identified through the Mayo Clinic Biobank [25], which enrolled 56,959 adults between 2009 and 2016, mainly from primary care clinics [26]. At enrollment, participants provided blood samples and completed a health history questionnaire. MBL screening was performed in a subset of Biobank participants, as described previously [6,10,11,27]. Participants aged 40 years or older, with no history of hematological cancer, and with available stored peripheral blood mononuclear cells (PBMCs), were eligible for MBL screening.

Patients with CLL were identified from the Mayo Clinic CLL Clinical Database [22,28,29], which has prospectively collected data since 1995 on patients seen in the Division of Hematology at Mayo Clinic, Rochester, MN, who agreed to the research use of their medical records [29]. Patients who consented to participate and provided a blood specimen within two years of their CLL diagnosis were included. Characteristics of the study groups are shown in Supplementary Table S1.

### 2.2. MBL Screening

Details of the MBL screening strategy in the Mayo Clinic Biobank have been reported previously [6,9,10,11,22]. In brief, stored PBMCs were evaluated with an eight-color flow cytometry assay (CD38, CD45, kappa, lambda, CD19, CD23, CD5, and CD20) that detects clonal B cells at a level of 0.005%, acquiring approximately 500,000 events per sample. Each MBL case was reviewed and confirmed by a Mayo Clinic hematopathologist with expertise in lymphoid malignancies. MBL clones were classified by immunophenotype as CLL-type (CD5+, CD20dim/CD20+), or non–CLL-type (CD5−). Individuals included herein were those who screened negative for MBL (MBL-negative) and those with LC-MBL (defined as the proportion of clonal B cells out of total B cells < 85%) with immunophenotype as CLL-type. Individuals with HC-MBL or another immunophenotype were excluded.

### 2.3. Genotyping

Genotyping was performed using Illumina arrays (Infinium OmniExpress or Global Screening Array) or by genotyping by sequencing at the Regeneron Genetics Center. DNA was extracted from whole blood. Quality control followed previously described procedures [22]. SNPs that were monomorphic, had call rates < 95%, or showed extreme deviation from Hardy–Weinberg equilibrium (*p* < 1.0 × 10^−5^) were removed, and individuals with sex discordance or a genotyped relative were excluded. Samples with call rates below 98% were removed; genotype concordance across platforms exceeded 99%. We evaluated the 41 SNPs reported in the largest GWAS of CLL to date [15].

### 2.4. Statistical Analysis

We performed three case-control analyses. The first compared individuals screened with LC-MBL to those who screened negative for MBL (MBL-negative) to identify variants associated with LC-MBL risk. Second, because CLL is always preceded by LC-MBL, we compared patients with CLL to individuals with LC-MBL to identify variants that may be markers of progression from LC-MBL to CLL, herein labeled as progression. We considered this analysis to be an indirect proxy for a longitudinal study that follows individuals with LC-MBL until progression to CLL or study end date. Finally, we compared patients with CLL to MBL-negative individuals to confirm the associations of the 41 SNPs with CLL risk. Logistic regression was used to estimate odds ratios (ORs) and 95% confidence intervals (CIs), with SNPs coded additively for the risk allele. All models were adjusted for age, sex, and the first ten principal components to account for population stratification. To test whether the OR for each SNP differed between the first two analyses, we used the Wald test for the difference in log ORs; *p*-values were adjusted using the Benjamini–Hochberg false discovery rate (FDR) across all 41 SNPs. SNPs with FDR < 0.05 and non-overlapping 95% CIs were considered to differ between analyses. Characteristics of the three groups were summarized using medians and percentages. Age was defined as age at MBL screening for Biobank participants and age at diagnosis for patients with CLL.

### 2.5. Polygenic Risk Score

As previously published [21,22,23], the CLL-PRS was calculated for each individual as a weighted sum of the CLL-susceptibility risk alleles, using weights derived from the largest GWAS of CLL to date [15] (Supplementary Table S2). The CLL-PRS was evaluated as a continuous variable and in quintiles, with the middle quintile as the reference, as it contains the most common PRS value in the general population [22]. Quintiles of the CLL-PRS41 were defined using previously published thresholds derived from 7983 controls in the NHL GWAS [21] which allows comparison with prior studies. Separately, in an exploratory analysis, we constructed a second score (CLL-PRS20) from the 20 of the 41 SNPs that were associated with progression (CLL vs. LC-MBL, *p* < 0.05). Quintiles of the CLL-PRS20 were defined using cut points derived from the distribution of the score among all MBL-negative individuals, given no published reference thresholds exist for this score. Analyses were performed using PLINK 1.9 [30] and R v4.4.1, and figures were produced using Python v3.7. Analysis code is available at https://github.com/rmwangi07/cll-mbl-susceptibility-continuum (accessed on 30 October 2026).

## 3. Results

Among the 2606 patients with CLL, 1601 individuals with LC-MBL, and 8322 MBL-negative individuals, the median age was 62 years, 65 years, and 61 years, respectively (Supplementary Table S1). CLL patients were more likely to be male (67%) than individuals with LC-MBL (50%) or MBL-negative individuals (36%).

**Figure 1:**
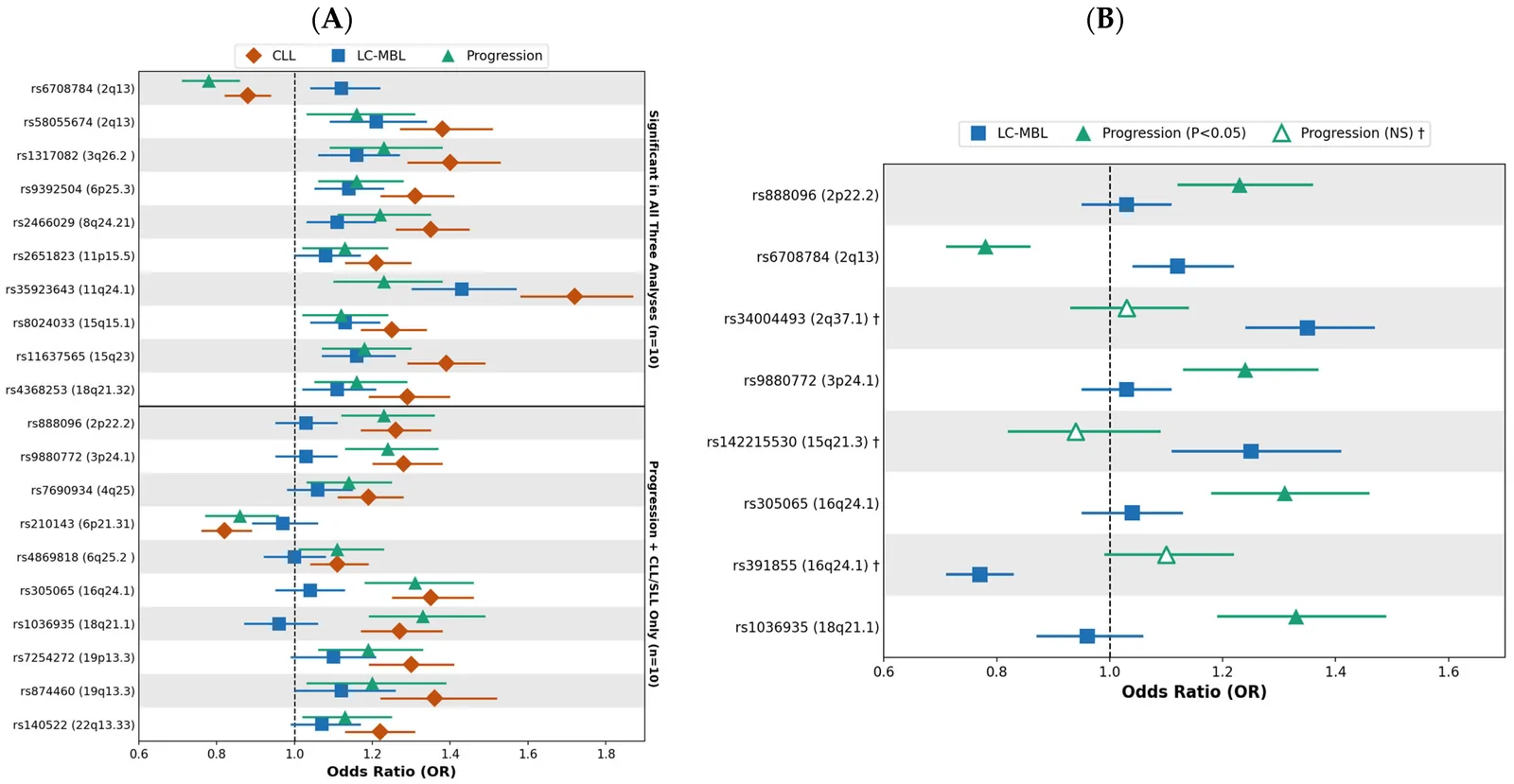
Associations of inherited CLL-risk variants with LC-MBL risk, progression, and CLL. All odds ratios (95% CI) are adjusted for age, sex, and principal components. (**A**) The 20 SNPs associated with progression (*p* < 0.05); 10 SNPs were also associated with LC-MBL (top) and 10 showed no evidence of association with LC-MBL (bottom); SNPs are ordered by chromosomal position within each subgroup. (**B**) The eight SNPs whose odds ratios differed between LC-MBL risk and progression (FDR < 0.05, non-overlapping 95% CIs required). Blue squares, LC-MBL versus MBL-negative; green triangles, CLL versus LC-MBL (progression); red diamonds, CLL vs. MBL-negative, filled triangles, *p* < 0.05 for progression; open triangles (†), *p* ≥ 0.05.

Of the 41 SNPs, 22 (54%) were associated with LC-MBL risk (all *p* < 0.05; Figure 1A and Supplementary Figure S1), similar to the 21 SNPs associated with overall MBL in our previous, smaller screening study [22].

In the case-control “progression” analyses of CLL versus LC-MBL, 20 SNPs (49%) were associated with progression at *p* < 0.05 (Figure 1A); 18 had positive associations and two had inverse. Of these 20 SNPs, 10 showed no evidence of an association with risk of LC-MBL, and 10 were associated with both LC-MBL and progression. All 20 SNPs were associated with CLL risk in this sample (all *p* < 0.05; Figure 1A). Because a SNP may be associated with both risk of LC-MBL and progression, but more strongly with one, we compared the ORs between the two analyses. Eight SNPs had significantly different ORs (FDR < 0.05) with non-overlapping 95% CIs (Figure 1B). Four SNPs had larger ORs for progression than for risk of LC-MBL (rs888096, 2p22.2; rs9880772, 3p24.1; rs305065, 16q24.1; rs1036935, 18q21.1), and two had larger ORs for LC-MBL than for progression (rs34004493, 2q37.1; rs142215530, 15q21.3). Two SNPs had associations in opposite directions; rs6708784 (2q13) was positively associated with LC-MBL (OR = 1.12, 95% CI: 1.04–1.22) but inversely associated with progression (OR = 0.78, 95% CI: 0.71–0.86; P for difference = 1.3 × 10^−8^), and rs391855 (16q24.1) was inversely associated with LC-MBL (OR = 0.77, 95% CI: 0.70–0.83), but positively associated with progression (OR = 1.10, 95% CI: 1.00–1.22; *p* for difference = 9.3 × 10^−8^).

Of the remaining 21 SNPs that were not statistically associated with progression, 18 had OR estimates for progression in the same direction as observed in the analyses for risk of CLL (i.e., CLL cases to MBL-negative individuals, Supplementary Figure S1); the lack of statistical significance in the progression analyses may be due to reduced sample size and thus reduced statistical power. Furthermore, of these 21 SNPs, 12 were statistically associated with LC-MBL risk, indicating these SNPs may play a role in clonal development (Supplementary Figure S1).

We next assessed the effect of the CLL-PRS. The distribution of the CLL-PRS derived from the 41 SNPs increased across the continuum from MBL-negative individuals to LC-MBL to CLL (Supplementary Figure S2). Using a sample size approximately four times larger than our prior screening study of 396 individuals with LC-MBL, we confirmed that the CLL-PRS was associated with risk of LC-MBL (OR = 1.77, 95% CI: 1.66–1.88, *p* = 3.7 × 10^−68^; Table 1) [21,22,23]. Compared to the middle quintile, individuals in the highest quintile of CLL-PRS had higher odds of LC-MBL compared to MBL-negative (OR = 2.15, 95% CI: 1.83–2.54) and those in the lowest quintile had reduced odds (OR = 0.55, 95% CI: 0.45–0.68). In the CLL vs. LC-MBL analyses, the CLL-PRS was also associated with progression (OR = 1.45, 95% CI: 1.35–1.56, *p* = 8.1 × 10^−24^; Table 1). Compared with the middle quintile, individuals in the highest CLL-PRS quintile had higher odds of CLL relative to LC-MBL (OR = 1.60, 95% CI: 1.32–1.93, *p* = 1.1 × 10^−6^), while those in the lowest quintile had reduced odds (OR = 0.63, 95% CI: 0.47–0.83, *p* = 9.2 × 10^−4^; Table 1). Finally, the CLL-PRS was associated with CLL (OR = 2.59, 95% CI: 2.44–2.74, *p* = 4.6 × 10^−220^), with ORs of 3.52 (95% CI: 3.06–4.06) for the highest quintile and 0.35 (95% CI: 0.28–0.43) for the lowest quintile compared with the middle quintile (Table 1).

In an exploratory analysis, we also evaluated the PRS of the 20 SNPs associated with progression (CLL-PRS20, Supplementary Table S3). The CLL-PRS20 was associated with progression (OR = 1.92, 95% CI: 1.74–2.13, *p* = 1.2 × 10^−35^). Compared with the middle quintile, individuals in the highest CLL-PRS20 quintile had higher odds of progression (OR = 1.81, 95% CI: 1.50–2.19, *p* = 9.1 × 10^−10^).

## 4. Discussion

Using a sample size over four times larger than any prior study of individuals screened for MBL, we have several notable findings on the impact of germline genetics across the CLL continuum. First, germline genetics plays a role in developing LC-MBL. The CLL-PRS was significantly associated with LC-MBL risk with an effect size of 1.8, consistent with our prior study of 396 individuals with LC-MBL and 2631 MBL-negative [22]. These findings indicate that the CLL-PRS may identify individuals at risk of LC-MBL. Moreover, just over 50% of the previously reported CLL susceptibility SNPs were statistically associated with LC-MBL. These findings suggest that these individual SNPs may mark or be involved in early clonal B-cell development rather than at the later CLL stage.

Next, germline genetics plays a role in progression. Twenty of the 41 SNPs were associated with progression from LC-MBL to CLL, and eight had ORs that differed between LC-MBL and progression, suggesting that the associations of these SNPs are not uniform across the etiologic continuum from LC-MBL to CLL. The most notable were the two SNPs with associations in opposite directions, rs6708784 at 2q13 and rs391855 at 16q24.1 (near the B-cell transcription factor *IRF8* involved in B-cell development) [15]. The biological basis for these opposing associations remains unclear. Interestingly, the risk allele of rs6708784 is associated with reduced expression of *BCL2L11* [31], which encodes the pro-apoptotic protein BIM. Reduced *BCL2L11* expression could decrease apoptosis and thereby promote the survival and persistence of abnormal B-cell clones. However, it remains unclear why the same allele is associated with reduced progression risk. Further studies are needed to help clarify these findings. Finally, the CLL-PRS was also associated with CLL compared to individuals with LC-MBL, suggesting its potential as a biomarker for progression in individuals with LC-MBL. CLL-PRS, along with acquired molecular features, such as clonal hematopoiesis [9] or somatic events [28,32], have been identified as potential biomarkers to identify individuals with MBL at risk for progression to CLL.

Our study has several strengths, including the large number of individuals screened for MBL using a sensitive flow cytometry assay and the inclusion of individuals who screened negative for MBL. Our study also has limitations. Although we identified 20 SNPs statistically associated with progression from LC-MBL to CLL, our case-control study design limits our ability to draw definitive inferences regarding progression. The ideal study would prospectively follow a cohort of individuals with LC-MBL for progression to CLL or study end date. However, given the relatively low rate of progression, our cohort of ~1600 LC-MBL will require extended follow-up to accrue enough CLL events for an adequately powered analysis. Replication in an independent cohort is also needed. The test for differences in ORs does not account for individuals with LC-MBL contributing to both analyses and may be anti-conservative. Genotypes were obtained from more than one platform, although concordance across platforms exceeded 99%. Individuals with HC-MBL, who have a higher risk of progression than those with LC-MBL [5,6], were not evaluated because of small numbers. The CLL-PRS20 analysis is exploratory and because the same individuals were used to identify the 20 SNPs and estimate their associations, these estimates are upwardly biased and require external validation.

Because LC-MBL is also associated with non-CLL lymphoid malignancies [6,9], future studies should evaluate whether the CLL-PRS, or PRSs for other lymphoma subtypes, are associated with these outcomes. Extending these analyses to outcomes after CLL diagnosis, such as time to first treatment and infection [12,13], would further characterize the role of inherited susceptibility across the continuum of B-cell malignancy.

## 5. Conclusions

In conclusion, although the CLL-PRS was associated with both development of LC-MBL and progression from LC-MBL to CLL, the inherited CLL susceptibility SNPs appear to have variable relationships with B-cell clone etiology and expansion. Eight of the 41 established SNPs associated with developing CLL can be separated into those associated with risk of LC-MBL and risk of progression from LC-MBL to CLL, supporting distinct genetic contributions to these two processes. Prospective follow-up studies of individuals with LC-MBL that integrate inherited risk, along with acquired molecular features, will be needed to determine whether these findings can inform predictions of progression to CLL.

## Figures and Tables

**Table 1:** Distribution and association of the CLL polygenic risk score (CLL-PRS41) across MBL-negative individuals, LC-MBL, and CLL. Quintile thresholds were derived from 7983 controls from the NHL GWAS [21]; Q3 is the reference. All models adjusted for age, sex, and principal components.

PRS	MBL-Negative_N=8322_	LC-MBL_N=1601_	CLL_N=2606_	CLL vs. LC-MBL			LC-MBL vs. MBL-Negative			CLL vs. MBL-Negative		
	N (%)	N (%)	N (%)	OR	95% CI	*p*-Value	OR	95% CI	*p*-Value	OR	95% CI	*p*-Value
**CLL-PRS41 Quintiles, n (%)**												
Q1 (<6.76)	1765 (21.2%)	169 (10.6%)	152 (5.8%)	0.63	0.47–0.83	9.2 × 10^−4^	0.55	0.45–0.68	1.6 × 10^−8^	0.35	0.28–0.43	3.7 × 10^−23^
Q2 (6.76–7.27)	1804 (21.7%)	240 (15.0%)	264 (10.1%)	0.79	0.62–1.00	0.050	0.78	0.65–0.94	9.6 × 10^−3^	0.60	0.50–0.72	2.8 × 10^−8^
Q3 (7.27–7.73)	1662 (20.0%)	286 (17.9%)	407 (15.6%)	Ref	Ref	Ref	Ref	Ref	Ref	Ref	Ref	Ref
Q4 (7.73–8.26)	1576 (18.9%)	363 (22.7%)	542 (20.8%)	1.06	0.86–1.31	0.580	1.39	1.17–1.65	2.1 × 10^−4^	1.45	1.24–1.69	2.7 × 10^−6^
Q5 (8.26+)	1512 (18.2%)	543 (33.9%)	1239 (47.6%)	1.60	1.32–1.93	1.1 × 10^−6^	2.15	1.83–2.54	4.2 × 10^−20^	3.52	3.06–4.06	2.7 × 10^−68^
Continuous	—	—	—	1.45	1.35–1.56	8.1 × 10^−24^	1.77	1.66–1.88	3.7 × 10^−68^	2.59	2.44–2.74	4.6 × 10^−220^

## Data Availability

Analysis code is available at https://github.com/rmwangi07/cll-mbl-susceptibility-continuum (accessed on 30 October 2026). Summary statistics for the 41 variants are provided in the Supplementary Materials. Individual-level genotype and clinical data are not publicly available because of participant consent and privacy restrictions; requests can be made to the Mayo Clinic Biobank through its data access process.

## Supplementary Materials

The following supporting information can be downloaded at: https://www.mdpi.com/article/10.3390/cancers18213556/s1, Figure S1: The 21 inherited CLL-risk variants that did not reach *p* < 0.05 for progression from LC-MBL to CLL; Figure S2: Distribution of the CLL-PRS41 across MBL-negative individuals, LC-MBL, and CLL; Table S1: Characteristics of MBL-negative individuals, individuals with LC-MBL, and patients with CLL; Table S2: The 41 CLL susceptibility SNPs, risk alleles, and weights used to construct the CLL-PRS41; Table S3: Distribution and association of the CLL polygenic risk score (CLL-PRS20) across MBL-negative individuals, LC-MBL, and CLL. References [15,21] are cited in the Supplementary Materials.
