# Supplementary material for: Associations of Chronic Lymphocytic Leukemia (CLL) Susceptibility Variants Across the CLL Etiologic Continuum
Source: Cancers (Basel). 2026 Nov 5;18(21):3556. doi: 10.3390/cancers18213556 (PMC13761904; doi:10.3390/cancers18213556)
Supplement: Supplementary file 1 [file cancers-18-03556-s001.zip › cancers-4661933-supplementary.pdf]

Supplementary Materials

Associations of chronic lymphocytic leukemia (CLL) susceptibility variants across the CLL etiologic continuum

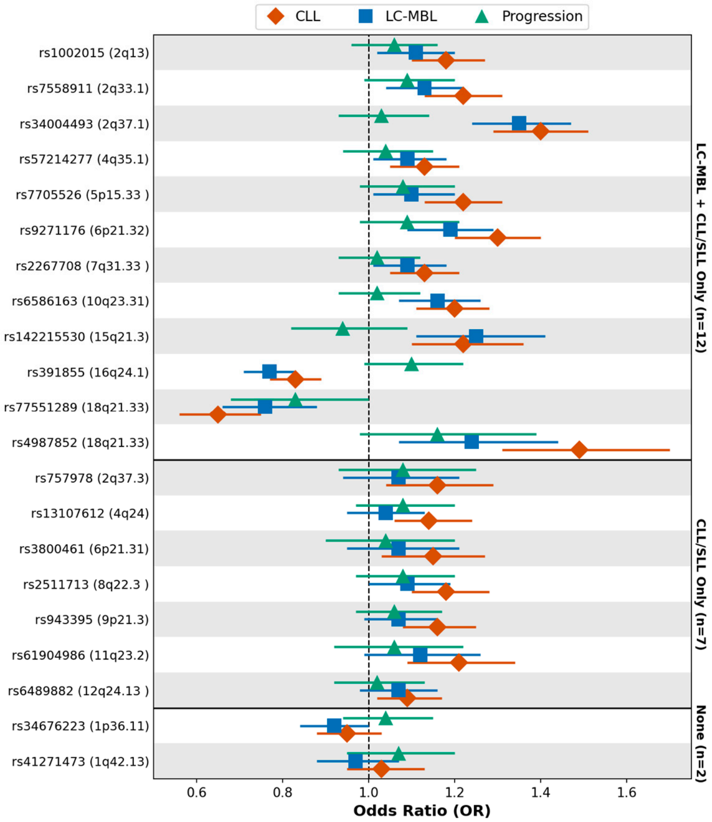

Supplementary Figure S1. The 21 inherited CLL-risk variants that did not reach P<0.05 for progression from LC-MBL to CLL. Odds ratios (95% CI) across the same three case-control analyses shown in Figure 1A, adjusted for age, sex and principal components, with SNPs ordered by chromosomal position within each subgroup; 12 associated with both LC-MBL and CLL but not progression (top), 7 associated with CLL only (middle), and 2 not associated in any analysis (bottom).

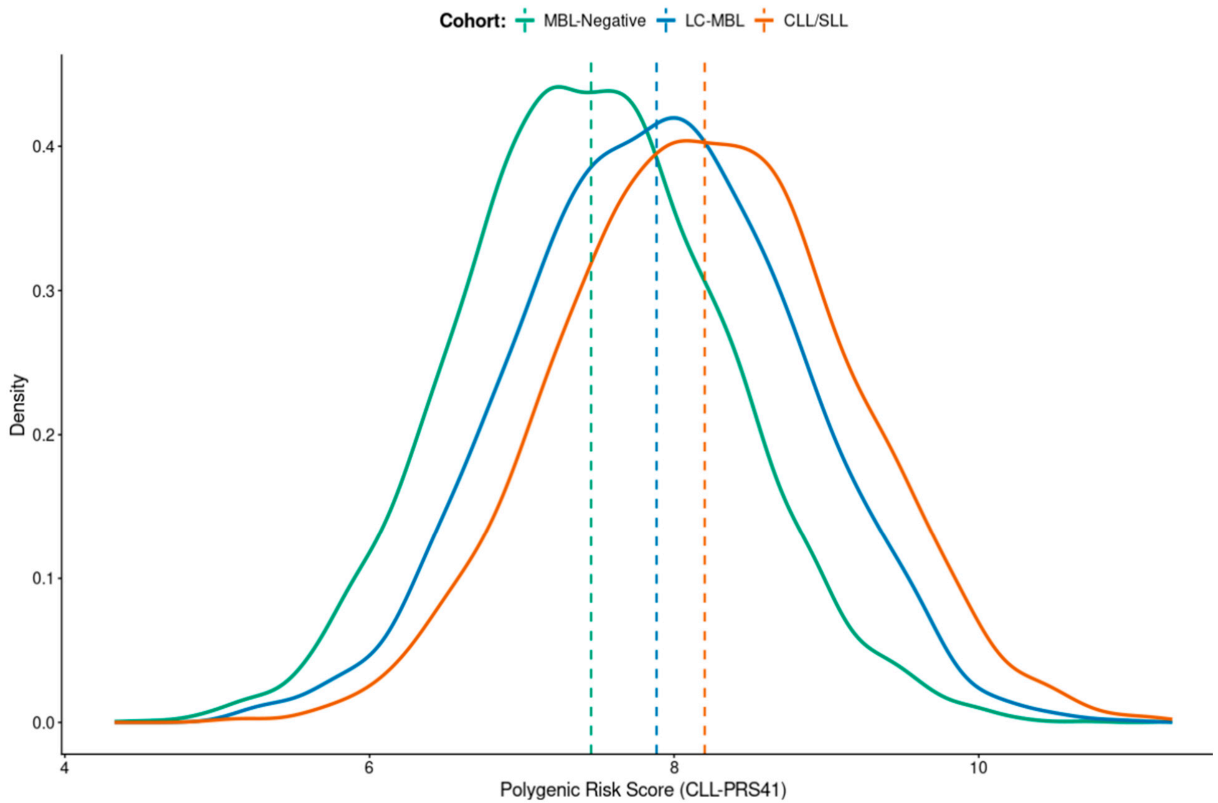

Supplementary Figure S2. Distribution of the CLL-PRS41 across MBL-negative individuals (N=8,322), LC-MBL (N=1,601), and CLL (N=2,606). Dashed lines indicate group means

Supplementary Table S1. Characteristics of MBL-negative individuals, individuals with LC-MBL, and patients with CLL.

Characteristic	MBL-negative (N = 8,322)	LC-MBL (N = 1,601)	CLL (N = 2,606)
**Age, years, median (Q1, Q3)**	**61 (52, 68)**	**65 (57, 72)**	**62 (54, 70)**
Missing	81	22	149
**Age group, years, n (%)**			
<55	2,810 (34%)	294 (19%)	658 (27%)
55–64	2,523 (31%)	454 (29%)	767 (31%)
65–74	2,058 (25%)	565 (36%)	707 (29%)
≥75	850 (10%)	266 (17%)	325 (13%)
Missing	81	22	149
**Sex, n (%)**			
Male	2,980 (36%)	795 (50%)	1,738 (67%)
Female	5,307 (64%)	797 (50%)	867 (33%)
Missing	35	9	1
**Self-reported race, n (%)**			
European	7,822 (94%)	1,547 (97%)	2,345 (90%)
Non-European	500 (6.0%)	54 (3.4%)	261 (10%)

Supplementary Table S1. Age is age at MBL screening for Mayo Clinic Biobank participants and age at diagnosis for patients with CLL. Percentages are of non-missing values. Q1, Q3, first and third quartiles.

Supplementary Table S2. The 41 CLL susceptibility SNPs, risk alleles and weights used to construct the CLL-PRS41.

Locus	Nearest gene(s)	SNP	Risk allele	OR (95% CI)	P	Weight (ln OR)
1p36.11	MDS2	rs34676223	C	1.19 (1.14–1.25)	5.04×10^{-13}	0.1740
1q42.13	RHOU	rs41271473	G	1.19 (1.13–1.26)	1.06×10^{-10}	0.1740
2p22.2	QPCT, PRKD3	rs888096	A	1.15 (1.09–1.21)	5.20×10^{-8}	0.1398
2q13	ACOXL, BCL2L11	rs1002015	C	1.30 (1.23–1.37)	2.23×10^{-23}	0.2624
2q13	ACOXL, BCL2L11	rs58055674	C	1.41 (1.32–1.50)	2.02×10^{-27}	0.3436
2q13	ACOXL, BCL2L11	rs6708784	G	1.30 (1.24–1.37)	2.67×10^{-25}	0.2624
2q33.1	CASP10, CASP8	rs7558911	A	1.18 (1.12–1.24)	5.05×10^{-11}	0.1655
2q37.1	SP110, SP140	rs34004493	G	1.39 (1.31–1.47)	3.67×10^{-32}	0.3293
2q37.3	FARP2	rs757978	T	1.29 (1.20–1.39)	5.80×10^{-11}	0.2546
3p24.1	EOMES	rs9880772	A	1.16 (1.11–1.22)	1.91×10^{-9}	0.1484
3q26.2	MYNN, TERC	rs1317082	A	1.19 (1.12–1.26)	5.77×10^{-9}	0.1740
4q24	BANK1	rs13107612	C	1.17 (1.11–1.22)	1.37×10^{-10}	0.1570
4q25	LEF1	rs7690934	C	1.16 (1.11–1.22)	6.08×10^{-9}	0.1484
4q35.1	LOC728175	rs57214277	T	1.13 (1.08–1.18)	3.69×10^{-8}	0.1222
5p15.33	TERT	rs7705526	A	1.18 (1.12–1.25)	5.90×10^{-10}	0.1655
6p25.3	IRF4	rs9392504	A	1.33 (1.26–1.40)	9.81×10^{-29}	0.2852
6p21.32	HLA	rs9271176	G	1.29 (1.22–1.36)	3.16×10^{-20}	0.2546

Locus	Nearest gene(s)	SNP	Risk allele	OR (95% CI)	P	Weight (ln OR)
6p21.31	BAK1	rs210143	C	1.26 (1.19–1.33)	5.77×10^{-16}	0.2311
6p21.31	C6orf106	rs3800461	C	1.20 (1.13–1.28)	1.97×10^{-8}	0.1823
6q25.2	IPCEF1	rs4869818	G	1.15 (1.09–1.21)	4.11×10^{-8}	0.1398
7q31.33	POT1	rs2267708	T	1.16 (1.10–1.22)	8.55×10^{-9}	0.1484
8q22.3	ODF1	rs2511713	G	1.17 (1.10–1.23)	6.04×10^{-8}	0.1570
8q24.21	POU5F1B	rs2466029	G	1.23 (1.17–1.30)	7.47×10^{-16}	0.2070
9p21.3	CDKN2B-AS1	rs1679013	C	1.16 (1.10–1.22)	2.17×10^{-8}	0.1484
10q23.31	ACTA2, FAS	rs6586163	A	1.23 (1.17–1.29)	1.14×10^{-15}	0.2070
11p15.5	C11orf21, TSPAN32	rs2651823	A	1.18 (1.13–1.25)	5.24×10^{-11}	0.1655
11q23.2	TMPRSS5	rs61904987	T	1.24 (1.16–1.32)	2.46×10^{-11}	0.2151
11q24.1	SCN3B, GRAMD1B	rs35923643	G	1.63 (1.53–1.72)	4.26×10^{-58}	0.4886
12q24.13	OAS3	rs6489882	G	1.16 (1.10–1.22)	4.76×10^{-8}	0.1484
15q15.1	BMF, BUB1B	rs8024033	C	1.26 (1.20–1.32)	7.13×10^{-19}	0.2311
15q21.3	RFX7, NEDD4	rs142215530	G	1.39 (1.29–1.50)	2.46×10^{-18}	0.3293
15q23	RPLP1	rs11637565	G	1.35 (1.28–1.42)	1.96×10^{-31}	0.3001
16q24.1	IRF8	rs305065	C	1.16 (1.10–1.22)	7.57×10^{-8}	0.1484
16q24.1	IRF8	rs391855	A	1.34 (1.27–1.41)	1.25×10^{-28}	0.2927
18q21.1	CXXC1	rs1036935	A	1.15 (1.10–1.21)	3.27×10^{-8}	0.1398
18q21.32	PMAIP1	rs4368253	C	1.17 (1.11–1.24)	1.26×10^{-8}	0.1570
18q21.33	BCL2	rs77551289	A	1.37 (1.25–1.50)	1.83×10^{-11}	0.3148
18q21.33	BCL2	rs4987852	C	1.32 (1.20–1.44)	4.66×10^{-9}	0.2776
19p13.3	ZBTB7A	rs7254272	A	1.17 (1.10–1.73)	4.67×10^{-8}	0.1570
19q13.3	PRKD2, STRN4	rs874460	C	1.24 (1.15–1.34)	3.37×10^{-8}	0.2151
22q13.33	NCAPH2	rs140522	T	1.15 (1.10–1.20)	2.70×10^{-9}	0.1398

Supplementary Table S2. Per-allele ORs are those reported by Law et al.[15] and used to construct the CLL-PRS by Kleinstern et al.[21]; the CLL-PRS41 is the weighted sum of risk-allele counts, with weights equal to the natural log of each OR. rs13107612 is a proxy for rs71597109 (4q24). OR, odds ratio; CI, confidence interval. In this study, rs1679013 (9p21.3) and rs61904987 (11q23.2) were analysed using the proxies rs943395 and rs61904986, respectively.

Supplementary Table S3. Distribution and association of the CLL polygenic risk score (CLL-PRS20) across MBL-negative individuals, LC-MBL, and CLL. Quintile thresholds were derived from the distribution of the score among all MBL negative individuals; Q3 is the reference. All models adjusted for age and sex.

PRS	MBL-Negative$_{N=8,322}$	LC-MBL$_{N=1,601}$	CLL$_{N=2,606}$	CLL vs. LC-MBL			LC-MBL vs. MBL-Negative			CLL vs. MBL-Negative		
	N (%)	N (%)	N (%)	OR	95% CI	P-Value	OR	95% CI	P-Value	OR	95% CI	P-Value
**CLL-PRS20 Quintiles, n (%)**												
Q1 (<3.21)	1,810 (21.8%)	232 (14.5%)	173 (6.6%)	0.54	0.42 – 0.69	2.0×10^{-6}	0.66	0.55 – 0.80	1.5×10^{-5}	0.36	0.30 – 0.44	5.4×10^{-24}
Q2 (3.21–3.58)	1,695 (20.4%)	249 (15.6%)	249 (9.6%)	0.72	0.57 – 0.91	6.5×10^{-3}	0.77	0.64 – 0.93	6.8×10^{-3}	0.56	0.47 – 0.67	3.5×10^{-10}
Q3 (3.58–3.91)	1,627 (19.6%)	306 (19.1%)	436 (16.7%)	Ref	Ref	Ref	Ref	Ref	Ref	Ref	Ref	Ref
Q4 (3.91–4.30)	1,639 (19.7%)	352 (22.0%)	600 (23.0%)	1.24	1.01 – 1.52	0.041	1.16	0.97 – 1.37	0.100	1.41	1.21 – 1.64	8.0×10^{-6}
Q5 (4.30+)	1,548 (18.6%)	462 (28.9%)	1,146 (44.0%)	1.81	1.50 – 2.19	9.1×10^{-10}	1.64	1.39 – 1.93	4.5×10^{-9}	2.90	2.53 – 3.34	3.0×10^{-50}
Continuous	—	—	—	1.92	1.74 – 2.13	1.2×10^{-35}	1.64	1.51 – 1.79	1.1×10^{-30}	3.11	2.88 – 3.36	6.8×10^{-178}

References

1 Marti, G.E.; Rawstron, A.C.; Ghia, P.; Hillmen, P.; Houlston, R.S.; Kay, N.; Schleinitz, T.A.; Caporaso, N.; International Familial CLL Consortium. Diagnostic criteria for monoclonal B-cell lymphocytosis. *Br. J. Haematol.* **2005**, *130*, 325–332.

2 Landgren, O.; Albitar, M.; Ma, W.; Abbasi, F.; Hayes, R.B.; Ghia, P.; Marti, G.E.; Caporaso, N.E. B-cell clones as early markers for chronic lymphocytic leukemia. *N. Engl. J. Med.* **2009**, *360*, 659–667.

3 Slager, S.L.; Lanasa, M.C.; Marti, G.E.; Achenbach, S.J.; Camp, N.J.; Abbasi, F.; Kay, N.E.; Vachon, C.M.; Cerhan, J.R.; Johnston, J.B.; et al. Natural history of monoclonal B-cell lymphocytosis among relatives in CLL families. *Blood* **2021**, *137*, 2046–2056.

4 Alaggio, R.; Amador, C.; Anagnostopoulos, I.; Attygalle, A.D.; Araujo, I.B.O.; Berti, E.; Bhagat, G.; Borges, A.M.; Boyer, D.; Calaminici, M.; et al. The 5th edition of the World Health Organization Classification of Haematolymphoid Tumours: Lymphoid Neoplasms. *Leukemia* **2022**, *36*, 1720–1748.

5 Rawstron, A.C.; Bennett, F.L.; O'Connor, S.J.; Kwok, M.; Fenton, J.A.; Plummer, M.; de Tute, R.; Owen, R.G.; Richards, S.J.; Jack, A.S.; et al. Monoclonal B-cell lymphocytosis and chronic lymphocytic leukemia. *N. Engl. J. Med.* **2008**, *359*, 575–583.

6 Slager, S.L.; Parikh, S.A.; Achenbach, S.J.; Norman, A.D.; Rabe, K.G.; Boddicker, N.J.; Olson, J.E.; Kleinstern, G.; Lesnick, C.E.; Call, T.G.; et al. Progression and survival of MBL: A screening study of 10 139 individuals. *Blood* **2022**, *140*, 1702–1709.

7 Rawstron, A.C.; Green, M.J.; Kuzmicki, A.; Kennedy, B.; Fenton, J.A.; Evans, P.A.; O'Connor, S.J.; Richards, S.J.; Morgan, G.J.; Jack, A.S.; et al. Monoclonal B lymphocytes with the characteristics of "indolent" chronic lymphocytic leukemia are present in 3.5% of adults with normal blood counts. *Blood* **2002**, *100*, 635–639.

8 Nieto, W.G.; Almeida, J.; Romero, A.; Teodosio, C.; López, A.; Henriques, A.F.; Sánchez, M.L.; Jara-Acevedo, M.; Rasillo, A.; González, M.; et al. Increased frequency (12%) of circulating chronic lymphocytic leukemia-like B-cell clones in healthy subjects using a highly sensitive multicolor flow cytometry approach. *Blood* **2009**, *114*, 33–37.

9 Boddicker, N.J.; Shanafelt, T.D.; Parikh, S.A.; Rabe, K.G.; Griffin, R.; Norman, A.D.; Yao, Y.; Tian, S.; Ma, T.; O'Brien, D.R.; et al. CHIP amplifies the risk of lymphoid malignancies in individuals with monoclonal B-cell lymphocytosis (MBL). *Blood Cancer J.* **2025**, *15*, 195.

10 Vallejo, B.A.; Ansari, A.; Parikh, S.A.; Achenbach, S.J.; Rabe, K.G.; Norman, A.D.; Olson, J.E.; Kay, N.E.; Braggio, E.; Hanson, C.A.; et al. Risk of Incident Melanoma Among Individuals With Low-Count Monoclonal B-Cell Lymphocytosis. *J. Clin. Oncol.* **2024**, *42*, 4153–4162.

11 Shanafelt, T.D.; Kay, N.E.; Parikh, S.A.; Achenbach, S.J.; Lesnick, C.E.; Hanson, C.A.; Kleinstern, G.; Olson, J.E.; Norman, A.D.; Rabe, K.G.; et al. Risk of serious infection among individuals with and without low count monoclonal B-cell lymphocytosis (MBL). *Leukemia* **2021**, *35*, 239–244.

12 Agius, R.; Brieghel, C.; Andersen, M.A.; Pearson, A.T.; Ledergerber, B.; Cozzi-Lepri, A.; Louzoun, Y.; Andersen, C.L.; Bergstedt, J.; von Stemann, J.H.; et al. Machine learning can identify newly diagnosed patients with CLL at high risk of infection. *Nat. Commun.* **2020**, *11*, 363.

13 International CLL-IPI working group. An international prognostic index for patients with chronic lymphocytic leukaemia (CLL-IPI): A meta-analysis of individual patient data. *Lancet Oncol.* **2016**, *17*, 779–790.

14 Berndt, S.I.; Skibola, C.F.; Joseph, V.; Camp, N.J.; Nieters, A.; Wang, Z.; Cozen, W.; Monnereau, A.; Wang, S.S.; Kelly, R.S.; et al. Genome-wide association study identifies multiple risk loci for chronic lymphocytic leukemia. *Nat. Genet.* **2013**, *45*, 868–876.

15 Law, P.J.; Berndt, S.I.; Speedy, H.E.; Camp, N.J.; Sava, G.P.; Skibola, C.F.; Holroyd, A.; Joseph, V.; Sunter, N.J.; Nieters, A.; et al. Genome-wide association analysis implicates dysregulation of immunity genes in chronic lymphocytic leukaemia. *Nat. Commun.* **2017**, *8*, 14175.

16 Berndt, S.I.; Camp, N.J.; Skibola, C.F.; Vijai, J.; Wang, Z.; Gu, J.; Nieters, A.; Kelly, R.S.; Smedby, K.E.; Monnereau, A.; et al. Meta-analysis of genome-wide association studies discovers multiple loci for chronic lymphocytic leukemia. *Nat. Commun.* **2016**, *7*, 10933.

17 Di Bernardo, M.C.; Crowther-Swanepoel, D.; Broderick, P.; Webb, E.; Sellick, G.; Wild, R.; Sullivan, K.; Vijayakrishnan, J.; Wang, Y.; Pittman, A.M.; et al. A genome-wide association study identifies six susceptibility loci for chronic lymphocytic leukemia. *Nat. Genet.* **2008**, *40*, 1204–1210.

18 Crowther-Swanepoel, D.; Broderick, P.; Di Bernardo, M.C.; Dobbins, S.E.; Torres, M.; Mansouri, M.; Ruiz-Ponte, C.; Enjuanes, A.; Rosenquist, R.; Carracedo, A.; et al. Common variants at 2q37.3, 8q24.21, 15q21.3 and 16q24.1 influence chronic lymphocytic leukemia risk. *Nat. Genet.* **2010**, *42*, 132–136.

19 Slager, S.L.; Rabe, K.G.; Achenbach, S.J.; Vachon, C.M.; Goldin, L.R.; Strom, S.S.; Lanasa, M.C.; Spector, L.G.; Rassenti, L.Z.; Leis, J.F.; et al. Genome-wide association study identifies a novel susceptibility locus at 6p21.3 among familial CLL. *Blood* **2011**, *117*, 1911–1916.

20 Speedy, H.E.; Di Bernardo, M.C.; Sava, G.P.; Dyer, M.J.; Holroyd, A.; Wang, Y.; Sunter, N.J.; Mansouri, L.; Juliusson, G.; Smedby, K.E.; et al. A genome-wide association study identifies multiple susceptibility loci for chronic lymphocytic leukemia. *Nat. Genet.* **2014**, *46*, 56–60.

21 Kleinstern, G.; Camp, N.J.; Goldin, L.R.; Vachon, C.M.; Vajdic, C.M.; de Sanjose, S.; Weinberg, J.B.; Benavente, Y.; Casabonne, D.; Liebow, M.; et al. Association of polygenic risk score with the risk of chronic lymphocytic leukemia and monoclonal B-cell lymphocytosis. *Blood* **2018**, *131*, 2541–2551.

22 Kleinstern, G.; Weinberg, J.B.; Parikh, S.A.; Braggio, E.; Achenbach, S.J.; Robinson, D.P.; Norman, A.D.; Rabe, K.G.; Boddicker, N.J.; Vachon, C.M.; et al. Polygenic risk score and risk of monoclonal B-cell lymphocytosis in caucasians and risk of chronic lymphocytic leukemia (CLL) in African Americans. *Leukemia* **2022**, *36*, 119–125.

23 Kleinstern, G.; Slager, S.L. The inherited genetic contribution and polygenic risk score for risk of CLL and MBL: A narrative review. *Leuk. Lymphoma* **2023**, *64*, 788–798.

24 Crowther-Swanepoel, D.; Corre, T.; Lloyd, A.; Gaidano, G.; Olver, B.; Bennett, F.L.; Doughty, C.; Toniolo, D.; Caligaris-Cappio, F.; Ghia, P.; et al. Inherited genetic susceptibility to monoclonal B-cell lymphocytosis. *Blood* **2010**, *116*, 5957–5960.

25 Olson, J.E.; Ryu, E.; Johnson, K.J.; Koenig, B.A.; Maschke, K.J.; Morrisette, J.A.; Liebow, M.; Takahashi, P.Y.; Fredericksen, Z.S.; Sharma, R.G.; et al. The Mayo Clinic Biobank: A building block for individualized medicine. *Mayo Clin. Proc.* **2013**, *88*, 952–962.

26 Olson, J.E.; Ryu, E.; Hathcock, M.A.; Gupta, R.; Bublitz, J.T.; Takahashi, P.Y.; Bielinski, S.J.; St Sauver, J.L.; Meagher, K.; Sharp, R.R.; et al. Characteristics and utilisation of the Mayo Clinic Biobank, a clinic-based prospective collection in the USA: Cohort profile. *BMJ Open* **2019**, *9*, e032707.

27 Boddicker, N.J.; Mwangi, R.; Robinson, D.P.; Allmer, C.; Rosenthal, A.C.; Habermann, T.M.; Feldman, A.L.; Rimsza, L.M.; King, R.L.; Larson, M.C.; et al. Risk of lymphoid malignancy associated with cancer predisposition genes. *Blood Cancer J.* **2025**, *15*, 71.

28 Kleinstern, G.; Boddicker, N.J.; O'Brien, D.R.; Allmer, C.; Rabe, K.G.; Norman, A.D.; Griffin, R.; Yan, H.; Ma, T.; Call, T.G.; et al. Tumor mutational load is prognostic for progression to therapy among high-count monoclonal B-cell lymphocytosis. *Blood Adv.* **2024**, *8*, 2118–2129.

29 Parikh, S.A.; Rabe, K.G.; Kay, N.E.; Call, T.G.; Ding, W.; Schwager, S.M.; Bowen, D.A.; Conte, M.; Jelinek, D.F.; Slager, S.L.; et al. Chronic lymphocytic leukemia in young (≤ 55 years) patients: A comprehensive analysis of prognostic factors and outcomes. *Haematologica* **2014**, *99*, 140–147.

30 Chang, C.C.; Chow, C.C.; Tellier, L.C.; Vattikuti, S.; Purcell, S.M.; Lee, J.J. Second-generation PLINK: Rising to the challenge of larger and richer datasets. *Gigascience* **2015**, *4*, 7.

31 Speedy, H.E.; Beekman, R.; Chapaprieta, V.; Orlando, G.; Law, P.J.; Martín-García, D.; Gutiérrez-Abril, J.; Catovsky, D.; Beà, S.; Clot, G.; et al. Insight into genetic predisposition to chronic lymphocytic leukemia from integrative epigenomics. *Nat. Commun.* **2019**, *10*, 3615.

32 Abdelbaky, S.B.; Giacopelli, B.; Rabe, K.G.; Yamaguchi, K.; Wu, Y.Z.; Yan, H.; Shanafelt, T.D.; Parikh, S.A.; Ding, W.; Hampel, P.J.; et al. Prediction of outcomes for high-count monoclonal B lymphocytosis using an epigenetic and immunogenetic signature. *Blood* **2024**, *143*, 1752–1757.
